# Divergence of Fecal Microbiota and Their Associations With Host Phylogeny in Cervinae

**DOI:** 10.3389/fmicb.2018.01823

**Published:** 2018-08-30

**Authors:** Jiaying Li, Songping Zhan, Xuanzhen Liu, Qiang Lin, Jianping Jiang, Xiangzhen Li

**Affiliations:** ^1^Key Laboratory of Environmental and Applied Microbiology – Environmental Microbiology Key Laboratory of Sichuan Province, Chengdu Institute of Biology, Chinese Academy of Sciences, Chengdu, China; ^2^University of Chinese Academy of Sciences, Beijing, China; ^3^Chengdu Medical College, Chengdu, China; ^4^Chengdu Zoo, Chengdu Institute of Wildlife, Chengdu, China; ^5^Fujian Provincial Key Laboratory of Soil Environmental Health and Regulation, College of Resources and Environment, Fujian Agriculture and Forestry University, Fuzhou, China

**Keywords:** Cervinae, fecal microbiota, species specificity, host divergence, phylosymbiosis

## Abstract

Gastrointestinal microbiota may shape the adaptation of their hosts to different habitats and lifestyles, thereby driving their evolutionary diversification. It remains unknown if gastrointestinal microbiota diverge in congruence with the phylogenetic relationships of their hosts. To evaluate the phylosymbiotic relationships, here we analyzed the compositions of fecal microbiota of seven Cervinae species raised in the Chengdu Zoo. All sampled animals were kept in the same environmental condition and fed identical fodder for years. Results showed that Firmicutes and Bacteroidetes were dominant in their fecal microbiota. Even though some bacteria (e.g., Ruminococcaceae) were found to be common in the feces of all investigated species, some genera (e.g., *Sharpea* and *Succinivibrio*) were only observed in animals with particular digestive systems. As for the intraspecies variations of microbial communities, only a few operational taxonomic units (OTUs) were shared among replicates of the same host species although they accounted for most of the total abundance. Correlation was observed between the fecal microbiota divergence and host phylogeny, but they were not congruent completely. This may shed new light on the coevolution of host species and their microbiota.

## Introduction

Gastrointestinal microbiota may play important roles in food digestion, energy metabolism, immunity regulation, and in shaping behavioral phenotypes of animals (Warnecke et al., 2007; Tremaroli and Bäckhed, 2012; Chevalier et al., 2015; De Palma et al., 2015). Correlations have been reported between the host phylogenies and divergence in their gut microbiota (Ochman et al., 2010; Brucker and Bordenstein, 2012a; Brooks et al., 2016; Groussin et al., 2017). It implies that the divergence of gut microbiota may link to the diversification of their hosts through evolution (Delsuc et al., 2014). The codivergent history of hosts’ genotypes and their microbiota is termed as “phylosymbiosis” (Brucker and Bordenstein, 2012a,b). This pattern has been observed in many species from sponges to primates (Ochman et al., 2010; Phillips et al., 2012; Easson and Thacker, 2014; Moeller et al., 2014; Sanders et al., 2014). The selective mechanisms of hosts for maintaining species-specific microbiota were revealed in a reciprocal transplantation study (Rawls et al., 2006). Moreover, Li et al. (2017) reported that the differences of gut microbiota were positively correlated with host divergence time of Glires, thus speculating that gut microbiota may reflect divergent time of diverse hosts. Additionally, the study of Kohl et al. (2018a) showed that the pattern of phylosymbiosis was stabilized across different gastrointestinal tract regions, as well as in feces.

Multiple factors, such as diet, geographic distribution, and physiological condition, can influence alimentary tract microbiota (Ley et al., 2005, 2006, 2008; Benson et al., 2010; Zhang et al., 2010). Although gut microbiota of conspecifics are often similar (Ochman et al., 2010), these factors also lead to discordances between gut microbiota divergence and hosts’ phylogenies. Diet, a dominant factor shaping the gut microbiota, often relates to environmental sources. Dietary change can alter gut microbiota vastly and instantly in humans (David et al., 2014). By investigating 32 ruminant livestock species, Henderson et al. (2015) demonstrated that the variations in rumen microbiota were related to host species’ identity than to diet. A similar pattern was observed in mice (Carmody et al., 2015). Most previous studies on the evolution of gut microbiota were carried out in mammalian species from different areas without strict control of diet or environment (Ochman et al., 2010; Li et al., 2017). Controlled studies were only performed in a few species, such as *Peromyscus* deer mice, *Drosophila* flies, mosquitoes, and *Nasonia* wasps (Brucker and Bordenstein, 2012a, 2013; Brooks et al., 2016; Kohl et al., 2018a). Considering shared effects of host phylogeny and their dietary strategies, the relationship between host genetic diversification and the gastrointestinal microbiota remains unclarified (Ley et al., 2008; Muegge et al., 2011).

Mammalian herbivores have the highest net diversification rate than carnivores and omnivores (Martin and Maron, 2012; Price et al., 2012). They exhibit the highest microbial diversity among mammalian species (Ley et al., 2008; Pope et al., 2010). Ruminants, with around 200 species, occupy many different habitats (Hofmann, 1989; Hackmann and Spain, 2010). Microbiota in herbivorous alimentary tract deliver myriad services to their hosts, such as breakdown of recalcitrant plant fiber, detoxification of plant secondary compounds, and production of essential amino acids and vitamins (Muegge et al., 2011; Karasov and Douglas, 2013; LeBlanc et al., 2013; Kohl et al., 2014; Kohl and Dearing, 2016). Microbiota have been investigated in some host species of ruminants; however, they are documented insufficiently in many rare species (Li et al., 2014; Henderson et al., 2015; Delgado et al., 2017; Guan et al., 2017).

To control the dietary impact on gastrointestinal microbiota, we surveyed seven Cervinae species fed with the same fodder for years in the Chengdu Zoo. Because previous evidence showed that fecal data can represent a combination of microbial communities distributed throughout the gastrointestinal tract, we used fecal samples as proxies of intestinal tract samples (Eckburg et al., 2005). Additionally, considering its non-invasive property, fecal sampling is beneficial for endangered species (Hu et al., 2017). Herein, we selected Cervini and Muntiacini subfamilies in the clade Cervinae. Within Cervini, there are five extant genera, namely *Axis* (hog deer), *Cervus* (red deer and sika), *Rusa* (sambar), *Dama* (fallow deer), and *Elaphurus* (Père David’s deer). Tufted deer (*Elaphodus cephalophus*), belonging to *Elaphodus*, was selected to represent Muntiacini. We examined compositions and diversity of fecal microbiota in these animals. Then we assessed the relationship between hosts’ mitochondrial genetic distances and microbial community dissimilarity matrices. This cross-species examination of the linkage between the microbiota and their hosts’ phylogenies will reveal a species-specific signature of symbiotic bacteria. More importantly, Père David’s deer, hog deer, and sambar are evaluated as threatened species in the International Union for Conservation of Nature (IUCN) Red List (Extinct in the Wild, Endangered, and Vulnerable, respectively); however, there is little information about their gut microbiota. Our findings will extend the knowledge about these rare mammalians’ gastrointestinal microbiota, which ultimately will serve in their conservation or management.

## Materials and Methods

### Ethics Statement

Compliance with the Ethics Committee of the Chengdu Institute of Biology, Chinese Academy of Sciences, and the methods used in this study were carried out in accordance with the approved guidelines.

### Sample Collection

We collected samples on April 06, 2017. *Axis* (hog deer, *Axis porcinus*), *Cervus* (red deer, *Cervus elaphus*; sika, *Cervus nippon*), *Rusa* (sambar, *Rusa unicolor*), Dama (fallow deer, *Dama dama*), and Elaphurus (Père David’s deer, *Elaphurus davidianus*) in Cervini and *Elaphodus* (tufted deer, *Elaphodus cephalophus*) in Muntiacini (Muntiacini contains two genera, and only *Elaphodus* was investigated in this study) were raised in Chengdu Zoo, Sichuan Province of China. All animals lived in the same environment condition and consumed the same fodder for years (nutritional ingredients are shown in **Supplementary Table S1**). The adult animals were kept in enclosures. All of them were healthy and not injected with any antibiotics or other treatments in the past 6 months. We collected fresh samples immediately after excretion. For each species, three piles of fecal pellets were selected, and each pile was excreted by an individual. Fecal specimens were frozen at -20°C rapidly. Surface part of a fecal sample was scraped to escape contamination. Finally, all the samples were transferred and stored at -40°C.

### Reconstructing Phylogeny of Host Species

The molecular phylogeny was reconstructed using complete mitochondrial DNA sequences. Corresponding sequences were downloaded from GenBank (Accession number: HQ832482.1, AB245427.2, NC_031835.1, JN632629.1, JN399997.1, MF435989, DQ873526.1). All sequences were aligned by Clustalw2 (Larkin et al., 2007). Reindeer (*Rangifer tarandus*) was used as the outgroup taxon (Accession number: AB245426.1). Pairwise distances of the complete mitochondrial sequences between host species were generated by Bayesian inference (Ronquist et al., 2012).

### DNA Extraction, PCR Amplification, and Sequencing

Genomic DNA extraction of fecal samples was performed with MoBio DNeasy PowerSoil DNA isolation kit (Qiagen, Germany). DNA concentration was checked using NanoDrop 2000 Spectrophotometer (Thermo Scientific, United States). V4–V5 hypervariable region of bacterial 16S rRNA gene was amplified by pair primers (515F: 5-GTGYCAGCMGCCGCGGTA-3 and 909R: 5-CCCCGYCAATTCMTTTRAGT-3) (Tamaki et al., 2011). A unique barcode at the 5′ end of 515F primer was incorporated to distinguish each sample. For each sample, we conducted two PCR reactions. Each 25 μL PCR reaction system included 10 ng DNA template, 2.0 μM each primer, 1.5 mM MgCl_2_, 0.4 μM dNTPs, 0.25 U of Taq (TaKaRa, Dalian) and 1x reaction buffer. The PCR condition was composed of 3 min at 94°C, 30 cycles of 94°C for 30 s, 56°C for 30 s, 72°C for 30 s, and 10 min at 72°C. Blank controls were used in DNA extraction and PCR amplification, and no amplification band was observed. Finally, samples were sequenced using an Illumina MiSeq sequencer (MiSeq Reagent Kit V.2, 500 cycles) at Environmental Genomic Platform of Chengdu Institute of Biology. Because the negative controls did not show any PCR bands, we did not sequence blank controls although blank sequencing controls may be useful in determining possible contaminations. The raw sequences were deposited in NCBI Sequence Read Archive with accession number SRP142187.

### Bioinformatics Analysis

QIIME Pipeline^1^ (Version 1.7.0) was used to analyze raw reads (Caporaso et al., 2010). Reads were merged using FLASH-1.2.8 software (Magoc and Salzberg, 2011). Low-quality sequences, chimeras, mitochondria, and chloroplasts were removed. Then operational taxonomic units (OTUs) were clustered at a cutoff of 97% sequence identity using CD-HIT (Li and Godzik, 2006; Edgar et al., 2011). After filtering out singleton sequences, the number of sequences per sample was normalized to 9,683, and the rarefaction curves were generated. Alpha diversity indices (observed OTUs, Chao 1, Shannon, Simpson, and equitability index) were calculated. One-way ANOVA was performed to test the difference among host species. To identify core microbiota of each host species, the number of shared OTUs among all replications were calculated. The shared OTUs/sequences were shown as the proportion of total OTUs/sequences per species. The bioinformatics tool Tax4Fun was used to examine whether the microbiota of these animals exhibited different predicted functions (Aßhauer et al., 2015). UpSet plots were generated using UpSetR in R (Conway et al., 2017).

Both OTUs and phylogeny-based approaches were used to explore the relationship between fecal microbiota and their hosts. For an OTU-based method, we visualized a Jaccard dissimilarity matrix of each host species dataset using unweighted pair group approach with arithmetic mean (UPGMA) dendrogram. Host mitochondria sequence data were analyzed using maximum likelihood (ML) implemented in RaxmlGUI 1.3 (Silvestro and Michalak, 2012) and Bayesian inference (BI) using MrBayes 3.12 (Ronquist and Huelsenbeck, 2003). The phylogenetic tree was constructed using ML and BI methods. For ML analysis, the bootstrap consensus tree inferred from 1,000 replicates was used to represent the evolutionary history of the taxa analyzed. Branches corresponding to partitions reproduced in less than 70% of bootstrap replicates were collapsed (Hillis and Bull, 1993). For BI analysis, two independent runs with four Markov Chain Monte Carlo simulations were performed for ten million iterations and sampled every 1,000th iteration. The first 25% of samples were discarded as burn-in. Convergence of the Markov Chain Monte Carlo simulations was assessed using Tracer v.1.4^2^. The congruent results were shown in **Supplementary Figure S4**. To test the correlations between the fecal microbiota and their hosts’ phylogenies, we used Mantel test (with 9,999 iterations) to compare the distance matrix of host mitochondrial completed sequences and Jaccard dissimilarity matrix of fecal microbiota. We validated the comparison by calculating the Robinson–Foulds and Matching Cluster congruency scores as previously described (Brooks et al., 2016). Matching Cluster and Robinson–Foulds *p*-values were determined by the probability of 80,000 randomized bifurcating dendrogram topologies yielding equivalent or more congruent phylosymbiotic patterns than the microbiota dendrograms (Brooks et al., 2016). For a phylogeny-based approach, unweighted UniFrac matrix was produced through the QIIME pipeline and plotted in principal coordinates analysis (PCoA) (Lozupone and Knight, 2005). To further assess the effects of host phylogeny, unweighted UniFrac distance matrices of different hosts were compared using Analysis of Similarity (ANOSIM) (with 999 iterations). For the comparisons among microbial community structures, weighted UniFrac matrix was also calculated.

## Results

### Overall Microbial Community Compositions

Microbial community compositions of 21 fecal samples from six Cervini species (*A. porcinus*, *C. elaphus*, *C. nippon*, *R. unicolor*, *D. dama*, and *E. davidianus*) and one Muntiacini species (*E. cephalophus*) were obtained by MiSeq sequencing method. In total, 8,849 non-singleton OTUs (at 97% sequence identity) were identified in the datasets. The fitted OTU-level rarefaction curves of Good’s coverage index reached stable values for all samples (**Supplementary Figure S1**), showing that sequencing depth was enough to capture most of the microbial diversity.

**FIGURE 1:**
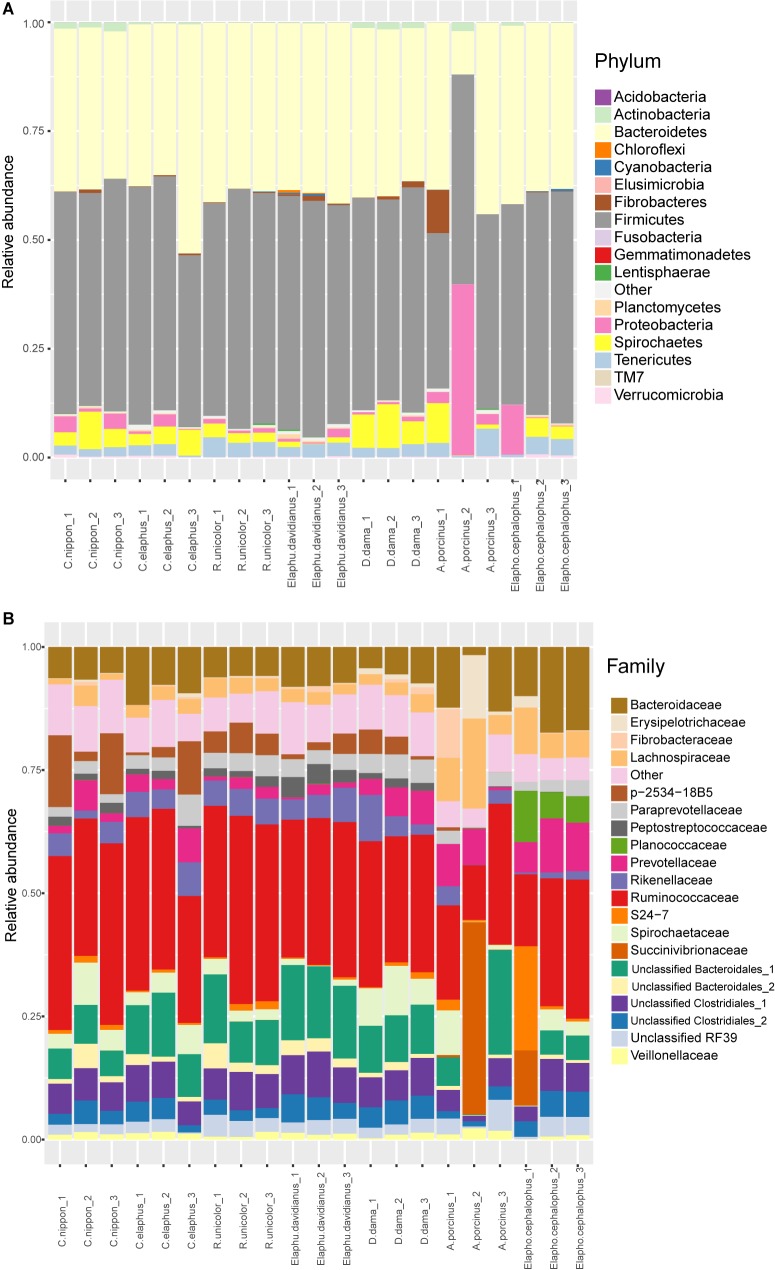
Taxonomic composition at the phylum **(A)** and family levels **(B)**.

Fecal microbiota were identified at different taxonomic levels. At phylum level, samples mainly contained Firmicutes (mean ± SD = 49.61% ± 5.04% of total sequences), Bacteroidetes (37.74% ± 7.43%), Spirochaetes (3.76% ± 3.03%), Proteobacteria (3.57% ± 8.06%), Tenericutes (2.68% ± 1.39%), and other phyla accounted for less than 1% of total sequences, e.g., Fibrobacteres 0.75% ± 2.13%, Actinobacteria, 0.68% ± 0.69%, Verrucomicrobia 0.25% ± 0.21%, and Planctomycetes 0.21% ± 0.22% (**Figure 1A**). Low variations among replications were observed in most deer species except for *A. porcinus*. At family level, Ruminococcaceae (28.50% ± 6.70%) was predominant in most samples (**Figure 1B**). The dominant taxa with the relative abundances greater than 5% included an unclassified family of Bacteroidales (9.20% ± 5.12%), Bacteroidaceae (8.43% ± 3.96%), and an unclassified family of Clostridiales (6.19% ± 1.78%). The relative abundance of Planococcaceae was much higher in Muntiacini (*Elaphodus cephalophus*) (5.33–10.38%) than that in Cervini species <0.14%). The taxonomic compositions for *A. porcinus* and *Elaphodus cephalophus* species at the family level may be doubtful because of the great variabilities among replicates. For instance, in certain replicates, the abundance of Succinivibrionaceae maximized at 39.05% in *A. porcinus* and 11.21% in *Elaphodus cephalophus*, whereas it ranged between 0 and 0.03% in other species. Most OTUs identified in our samples are related to strict or facultative anaerobic or facultative anaerobic microbes being representative members of gut microbiota. They are obviously different from the majority components of contamination (aerobic microbes) described by Salter et al. (2014). It may partially support the reliability of our sampling and sequencing procedures.

### The Inter- and Intra-Species Core Microbiota in Cervinae

**FIGURE 2:**
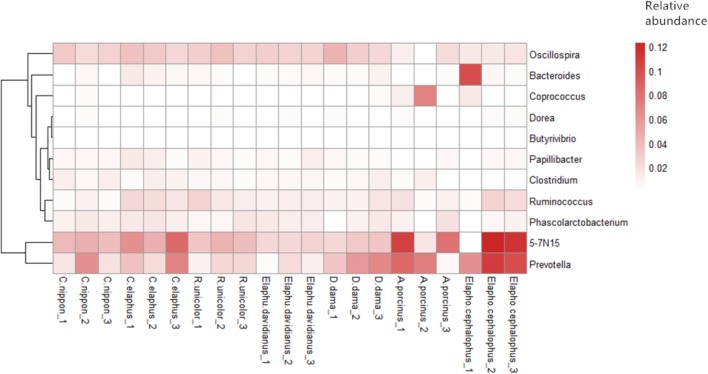
Relative abundance of annotated core genera.

Core genera affiliated to phylum Actinobacteria, Bacteroidetes, Firmicutes, Proteobacteria, Tenericutes, and Verrucomicrobia were further investigated across all samples. In addition, TM7, Chloroflexi, and Elusimicrobia served as species-specific core phyla (presented in all samples per species) with low relative abundances (0.04% ± 0.01%, 0.33% ± 0.28% and 0.02% ± 0.02%) in *C. nippon*, *Elaphurus davidianus*, and *D. dama*, respectively. Twenty-eight core genera (11 of them can be annotated at genus level) were identified almost within Firmicutes (19 genera), Bacteroidetes (7 genera), and Tenericutes (1 genus). Most OTUs (74.42% ± 9.23%) in each sample were affiliated to these genera. Spearman’s rank correlation coefficient was calculated to demonstrate the co-occurrence patterns among 11 annotated core genera. Our results indicated that patterns among core taxa of microbiota were not consistent in different host species (**Figure 2** and **Supplementary Figure S2**).

**FIGURE 3:**
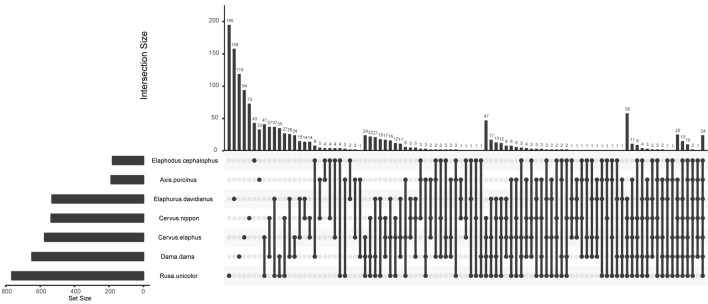
An Upset plot of host species-specific core OTUs. Set metadata of each host species was plotted to the left of the set size bar (charts). Dark circles indicated samples with containing accessions and connecting bar indicated multiple overlapping samples.

To identify inter- and intra-species core bacterial taxa, the shared and the host species-specific core OTUs were examined (**Table 1** and **Figure 3**). The shared OTUs among replicates of each species were relatively low (3.15–22.39% of total OTUs), especially for *Elaphodus cephalophus*. Nevertheless, for the majority of species, these shared OTUs contributed a large fraction of the total sequences (55.24%–77.00%), with the exceptions of *A. porcinus* (39.26% ± 13.14%) and *Elaphodus cephalophus* (27.16% ± 1.96%). Among 8,849 OTUs, 136 core OTUs of Muntiacini occurred in Cervini and 24 of them served as core OTUs across all species. Most (66.67%) of these common core OTUs of each species were from Ruminococcaceae family. Species-specific core OTUs were also assessed. For example, OTUs associated with *Succinivibrio* showed high abundances in *A. porcinus* (11.58%), whereas they were scarcely detected in other species (except for one sample of *Elaphodus cephalophus*). *Sharpea* had high prevalence and little variation in all individuals of *D. dama* (0.88%) and low abundance in a small number of individuals of other species (∼0.03%). The taxonomic assignments of core OTUs were summarized in **Supplementary Table S2**. Despite the low percentages of common core OTUs, the 274 KEGG pathways were discovered through predicted functional analysis of microbiota and 259 of them were present among all sampled animals (**Supplementary Figure S3** and **Supplementary Table S3**).

**Table 1:** Alpha-diversity estimates and percentages of shared microbiota among same species samples.

	Richness estimates		Diversity estimates			Core microbiota	
				
Species	Observed OTUs	Chao 1	Shannon	Simpson	Equitability	Shared OTUs (%)	Shared seqs (%)
*C. nippon*	1582 ± 222^a^	2686 ± 468^ab^	8.66 ± 0.38^a^	0.991 ± 0.003^a^	0.82 ± 0.02^a^	17.56	76.60 ± 8.19
*C. elaphus*	1755 ± 236^a^	2953 ± 374^ab^	9.09 ± 0.51^a^	0.994 ± 0.002^a^	0.84 ± 0.03^a^	17.48	64.03 ± 4.27
*R. unicolor*	1931 ± 98^a^	3141 ± 204^b^	9.39 ± 0.09^a^	0.995 ± 0.001^a^	0.86 ± 0.00^a^	22.39	77.00 ± 1.44
*Elaphurus davidianus*	1923 ± 155^a^	2994 ± 193^ab^	9.51 ± 0.23^a^	0.996 ± 0.001^a^	0.87 ± 0.01^a^	14.20	55.24 ± 2.11
*D. dama*	1703 ± 36^a^	2675 ± 51^ab^	9.11 ± 0.09^a^	0.995 ± 0.001^a^	0.85 ± 0.01^a^	21.25	76.80 ± 1.44
*A. porcinus*	1300 ± 472^a^	2202 ± 681^ab^	7.49 ± 1.71^a^	0.947 ± 0.063^a^	0.72 ± 0.13^a^	6.56	39.26 ± 13.14
*Elaphodus cephalophus*	1233 ± 471^a^	1793 ± 801^a^	8.09 ± 1.01^a^	0.986 ± 0.009^a^	0.79 ± 0.05^a^	3.15	27.16 ± 1.96


*One-way ANOVA was used to compare the differences of alpha-diversity indices across host species. Significant differences are indicated by different letters (*P* < 0.05) in the same column.*

### Comparisons of Gut Microbiota Among Cervinae Species

The variations of gut microbiota between and within each Cervinae species were reflected in the alpha diversity (**Table 1**). *R. unicolor* had the highest Chao 1 richness values (3,141 ± 204), whereas *A. porcinus* owned the highest variability in the replications (2,202 ± 681). In contrast, *Elaphodus cephalophus* possessed the lowest mean richness value (1,793 ± 801), whereas *D. dama* held the lowest variable richness value among the replications (2,675 ± 51). However, no significant differences were observed in terms of Shannon and Simpson indices.

**FIGURE 4:**
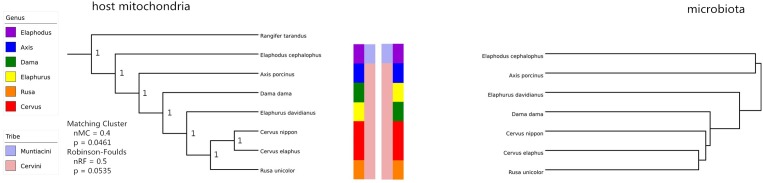
Mitochondrial completed sequences gene-based Cervinae phylogeny (left) and host microbiota based on OUT (right). Normalized Robinson–Foulds (RF) and normalized Matching Cluster (MC) metrics were determined as Brooks et al. (2016) described. Normalized metrics (nRF and nMC) scale from 0.0 (complete congruence) to 1.0 (complete incongruence).

**FIGURE 5:**
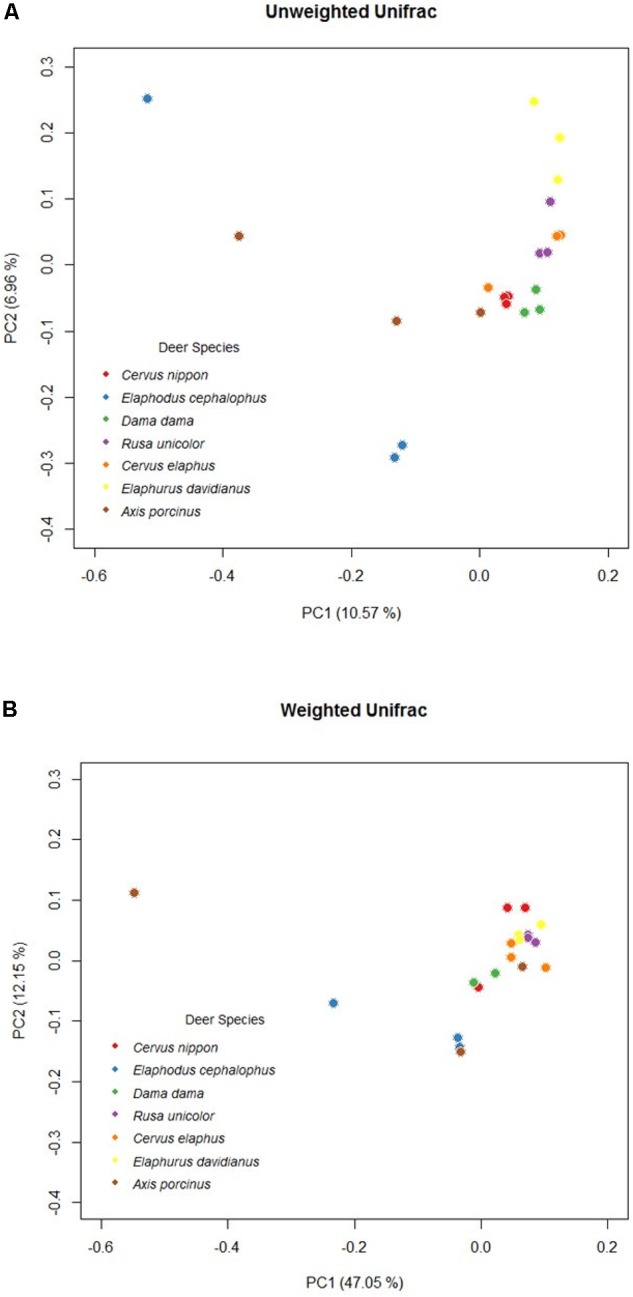
Principal coordinates plots based on OTU-level unweighted**(A)** weighted **(B)** UniFrac distance among Cervinae.

A remarkable overall correlation between fecal microbiota and their hosts’ phylogenies was observed based on Mantel test (*r* = 0.8025, *P* < 0.05). The significance of this pattern was validated using Robinson–Foulds and Matching Cluster analysis (nRF = 0.5, *P* = 0.0535; nMC = 0.4, *P* = 0.0461). Nevertheless, the hierarchical dendrogram of microbiota and their hosts’ phylogenies were not completely congruent (**Figure 4**). ANOSIM method identified significant differences of unweighted UniFrac distance matrices at tribe (*r* = 0.68, *P* < 0.05), genus (*r* = 0.60, *P* = 0.001), and species level (*r* = 0.52, *P* = 0.001). In addition, *Axis* (*r* = 0.80, *P* < 0.05) and *Elaphodus* (*r* = 0.40, *P* < 0.05) showed significant differences when compared with *Cervus*. The global differences in microbial community compositions were clearly visualized by PCoA of unweighted UniFrac distance matrix (**Figure 5A**). Higher community dispersions were detected in individuals of *Elaphodus cephalophus* and *A. porcinus*, and samples from these two species deviated from other five species. Weighted UniFrac distance matrix (including bacterial taxa abundance) revealed a similar pattern but with less variability among individuals, especially for Cervini species (expect *A. porcinus*) (**Figure 5B**).

## Discussion

Cervidae is the second most diverse subclade among terricolous artiodactyls. They natively inhabit from tropics to arctic regions and adapt themselves to various environments (Mattioli, 2011). We did the first study to investigate bacterial communities in closely related Cervinae species reared in the same environmental conditions. Our sample collection strategy provided a framework for characterizing host-bacterial generality and specificity in Cervinae species.

Our data revealed that the dominant phyla in these seven Cervidae species were composed of Firmicutes and Bacteroidetes, which play important roles in food fermentation (Ley et al., 2006, 2008; Durso et al., 2010; Li et al., 2014; Delgado et al., 2017; Hu et al., 2017). Low-abundant bacteria were affiliated to Spirochaetes, Fibrobacteres, Verrucomicrobia, Proteobacteria, Tenericutes, and Actinobacteria. These microbial components were also observed in other ruminants (Kong et al., 2010; Pope et al., 2012; Jami et al., 2013; Kittelmann et al., 2013; Pitta et al., 2014a,b). It seems that only a small number of bacterial phyla can adapt to the gut environment (Ley et al., 2006, 2008). At family level, as described in a previous study about moose (Ishaq and Wright, 2012), Peptococcaceae, Clostridiaceae, Succinivibrionaceae, and Lachnospiraceae were observed in the seven Cervinae species. These bacterial families are found in healthy rats rather than in their counterparts with irritable bowel syndrome (Nelson et al., 2011). This may indicate the health condition of these cervids in this experiment.

Interestingly, high levels of the variability were observed for some taxa (**Figure 1**). At phylum level, Bacteroidetes and Proteobacteria were evaluated as highly variable taxa. It was in agreement with the previous studies for cows, yaks, and humans (Arumugam et al., 2011; Jami and Mizrahi, 2012; Guo et al., 2015; Tang et al., 2017). Jami and Mizrahi (2012) revealed that the abundance of Bacteroidetes accounted for 26–70% of all reads, whereas the abundances of Proteobacteria ranged from 0.5% to 20% of all reads in some samples although all cows were fed in the same farm with same diet. Guo et al. (2015) also observed a significant fluctuation of the relative abundance of Proteobacteria among individual yaks though all yaks were male and lived in the same farm under similar nutritional conditions. At family level, Succinivibrionaceae and S24-7 were dominantly variable taxa. Tang et al. (2017) also observed the same pattern in cows that these taxa contribute to cow-to-cow variation under the same feeding regimen at the same farm. This may indicate that different enterotypes exist in cervids.

Important genera were identified owning cellulolytic (e.g., *Fibrobacter*, *Ruminococcus*, *Butyrivibrio*), lipolytic (e.g., *Anaerovibrio*), proteolytic (e.g., *Clostridium*, *Bacteroide*s), and amylolytic (e.g., *Prevotella*, *Bifidobacterium*) functions (Weimer, 1993; Warnecke et al., 2007; Brulc et al., 2009; Pitta et al., 2010; Dai et al., 2014; Nie et al., 2018). *Ruminococcus* contained almost all of the core OTUs shared among host species. *Ruminococcus* is well known for its fiber-degrading capability (Leatherwood, 1965). It is common and dominant in the gastrointestinal tracts of herbivores (Henderson et al., 2015). Apart from deer, *Ruminococcus* was observed in many other species, e.g., alpaca, cattle, goat, horse, sheep, pika, and rhinoceros (Dowd et al., 2008; Kim et al., 2011; Robert, 2012; Li et al., 2017; Neumann et al., 2017; O’Donnell et al., 2017; Kohl et al., 2018b). It may indicate the housekeeping functions of this genus. We also assessed the signature core taxa for each species from these 21 samples. *Sharpea azabuensis* owns rapid heterofermentative growths, and it plays an essential role in lactate production and utilization (Kamke et al., 2016). Despite some anomalies, *S. azabuensis* had higher relative abundance (0.88%) in *D. dama* than those in other host species (∼0.03%). A previous study showed that a smaller rumen size with a higher turnover rate may tend to select microorganisms that are capable of fast, heterofermentative growth on sugars (Bain et al., 2014; Goopy et al., 2014; Kittelmann et al., 2014). Thus, we speculated that *D. dama* might rely more on lactate heterofermentative metabolism than other examined species. A previous report revealed that the populations of *Succinivibrio* sp., *Eubacterium* sp., and *Robinsoniella* sp. correlate with digestion efficiency because of their potential metabolic capability (e.g., formate production, propionate synthesis, and syntrophic interactions with methanogens) (Hernandez-Sanabria et al., 2012). Our datasets showed that *A. porcinus* harbored high abundance of *Succinivibrio* (11.58%) and *Eubacterium* (0.028%) than other host species, which suggested that *A. porcinus* might have higher digestion efficiency.

The correlations among core bacterial genera were not completely congruent among different species. For instance, genera *Prevotella* and *Clostridium* showed markedly positive correlations between *D. dama* and *A. porcinus* but negative correlations between *R. unicolor* and *C. nippon*. It contrasted with the results of Li et al. (2017), which showed completely consistent co-occurrence patterns of core genera among eight Glires species. Because the digestive tracts of different host species differ in niche specificity and nutrient availability, taxa with positive or negative core bacteria correlation patterns may indicate that the co-occurrence of core genera is only achieved in some specific environments (Faust et al., 2012).

Although all hosts were raised in identical living conditions with the same fodder, shared OTUs among replicates were surprisingly low (3.15–22.39%), particularly in *A. porcinus* and *Elaphodus cephalophus* (6.56% and 3.15%, respectively). Besides, in terms of alpha-diversity indices, *D. dama* species harbored relatively constant bacterial diversity between the replicates, whereas *A. porcinus* and *Elaphodus cephalophus* had large intraspecies variations. A previous study elucidated that the hosts can select specific taxa (Smith et al., 2015), and the variations of microbiota existed among individuals of species or genotype (Smith et al., 2015). These observed intra- and inter-species variations on microbiota may serve as indicators of the ecological processes, which shape host-associated microbial community. Because host-associated microbial communities are shaped by both deterministic and stochastic processes, we can speculate that stochastic processes may play more important roles in shaping microbiota in *A. porcinus* and *Elaphodus cephalophus* species rather than that in *D. dama*. In addition, despite the low counts of shared OTUs among replications of each host-species, the shared OTUs accounted for most retrieved sequences (except *A. porcinus* and *Elaphodus cephalophus*). This was consistent with a previous study on surgeonfishes (Miyake et al., 2015). Despite the variations of community and structure, functional stability was observed. An increasing number of studies revealed that microbial taxa can be de-coupled with their function (Burke et al., 2011; Purahong et al., 2014; Louca et al., 2016; Bletz et al., 2017). Multiple microbial taxa selected by or adapted to a host may be functionally redundant in a host-associated community (Lozupone et al., 2012; Bletz et al., 2017). It must be noted that the functional prediction through a bioinformatics approach in this study was based on current database and the assumptions of functional equivalence of 16S gene matches. However, the results in our study may serve as a preliminary indication. Future analyses concerning metagenomic and metatranscriptomic approaches will be helpful to elucidate the interactions of host–microbiota, as well as microbial structure and function.

Although the distance matrices of hosts’ phylogenies were not strictly consistent with those of microbial compositions, a remarkable association between them was observed in this study. It is in line with the study of surgeonfishes (Miyake et al., 2015). Ley et al. (2008) demonstrated complete congruence between gut microbiota and host phylogeny at order level, whereas Roeselers et al. (2011) and Li et al. (2017) observed similar patterns at species and even subspecies level. These studies suggest that host phylogeny may be a driver shaping gut microbiota. However, because evidence showed that gastrointestinal microbiota can be influenced by multiple factors, such as diet, geographic distribution, and physiology, the intention to predict the divergent histories of hosts seems less convincing, especially among affinis species merely relying on dissimilarities among gut microbiota. In addition, there were substantial variations between replicates when the community compositions were considered rather than community structure. Because unweighted UniFrac analysis is sensitive to rare taxa and weighted UniFrac analysis is sensitive to abundant taxa, it is possible that the differences in bacterial communities among host species are mainly induced by rare taxa (Koren et al., 2013).

## Conclusion

This study provides the first investigation of fecal microbiota of Cervinae animals (including some threatened species) at different phylogenetic levels fed with same fodder. Results showed that Cervinae animals shared common fecal microbiota (e.g., Ruminococcaceae), but some bacterial genera (e.g., *Sharpea* and *Succinivibrio*) were associated only with particular digestion types. Because common and host-specific gastrointestinal microbiota were selected and maintained in Cervinae, the distance matrices of gastrointestinal microbiota and their hosts’ phylogenies were not completely congruent. To clarify the accurate relationship between gastrointestinal microbiota and their hosts’ phylogenies, a large sample size of both reared and wild populations with more information (e.g., gender, age, and body size) is needed.

## Author Contributions

JL and XiL designed the research. JL, SZ, XuL, QL, and JJ contributed to experimental work. JL performed the data analysis and wrote the manuscript. JL and XiL revised the manuscript.

## Conflict of Interest Statement

The authors declare that the research was conducted in the absence of any commercial or financial relationships that could be construed as a potential conflict of interest.

## Footnotes

1 http://qiime.org/

2 http://tree.bio.ed.ac.uk/software/tracer/

## Supplementary Material

FIGURE S1 OTU-level rarefaction curves of Goods coverage across all samples.

FIGURE S2 Co-occurrence patterns of core genera across the all host species **(A)** and within one host species **(B–H)**, as determined by Spearman’s rank correlation analysis.

FIGURE S3 UpsetR plot of functional prediction.

FIGURE S4 Host cladogram.

TABLE S1 Component of fodder.

TABLE S2 Intraspecies core microbiota in Cervinae.

TABLE S3 Pathways of functional prediction.
