# Supplementary figures and images for: Divergence of Fecal Microbiota and Their Associations With Host Phylogeny in Cervinae
Source: Front Microbiol. 2018 Aug 30;9:1823. doi: 10.3389/fmicb.2018.01823 (PMC6125396; doi:10.3389/fmicb.2018.01823)

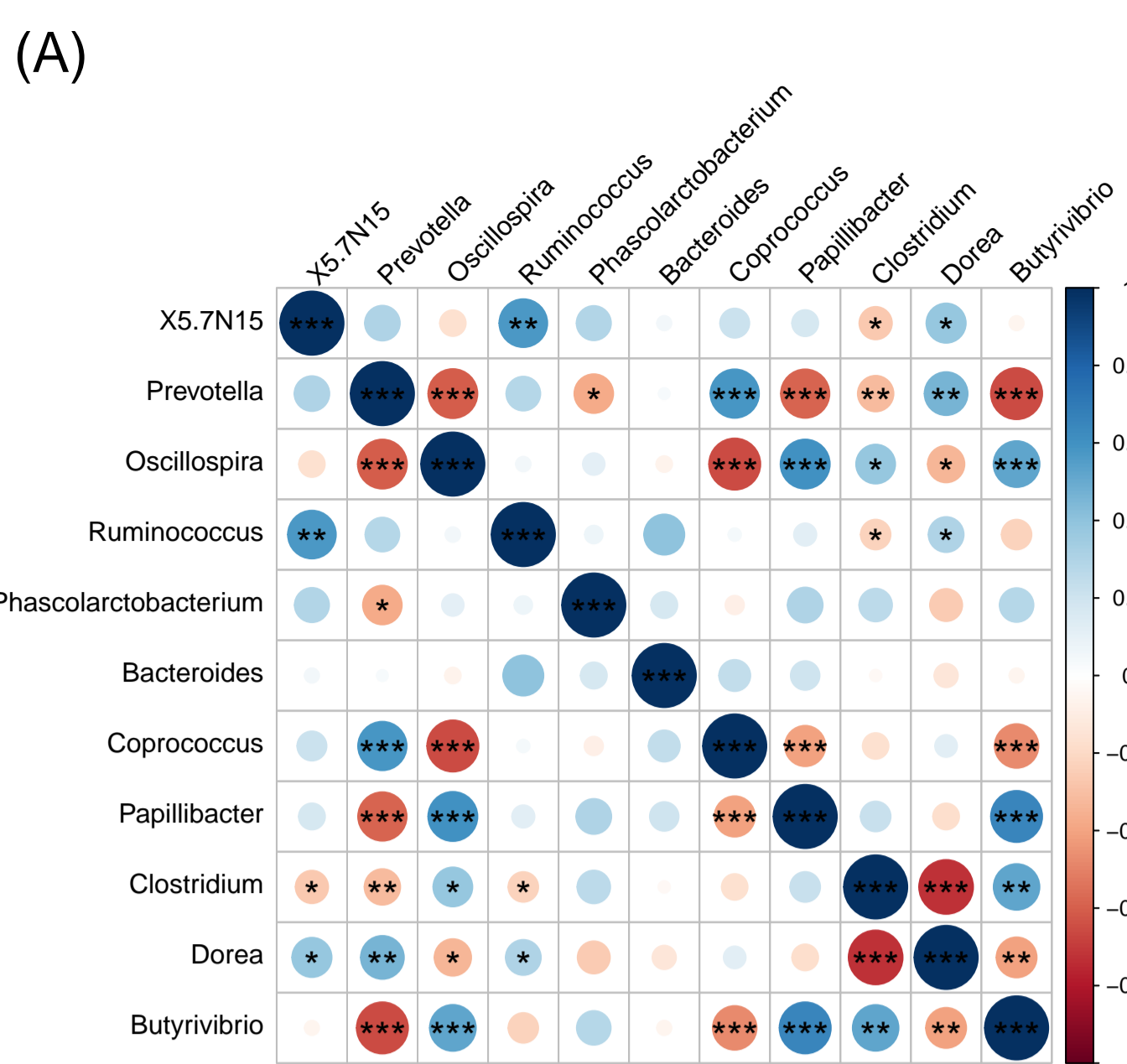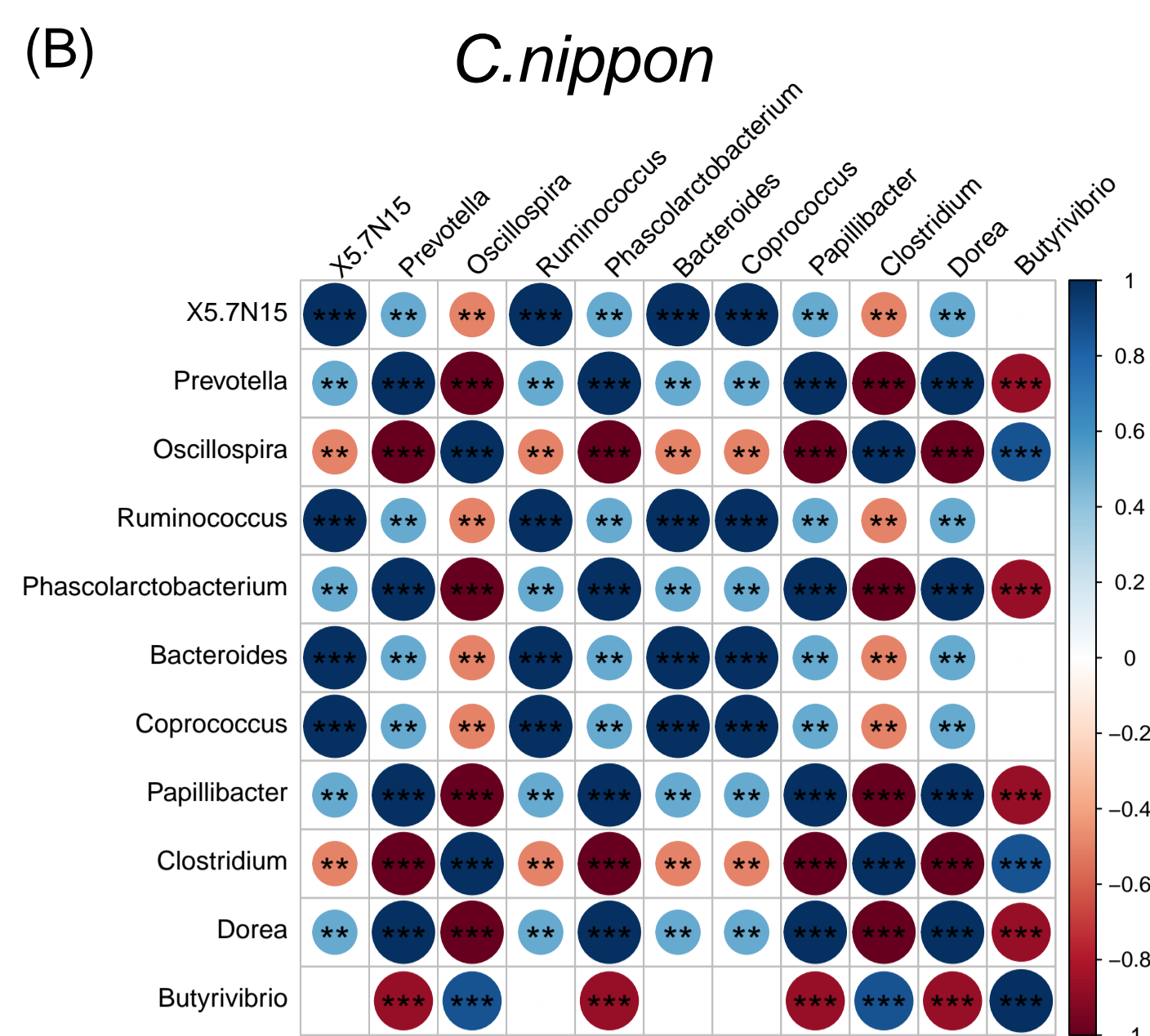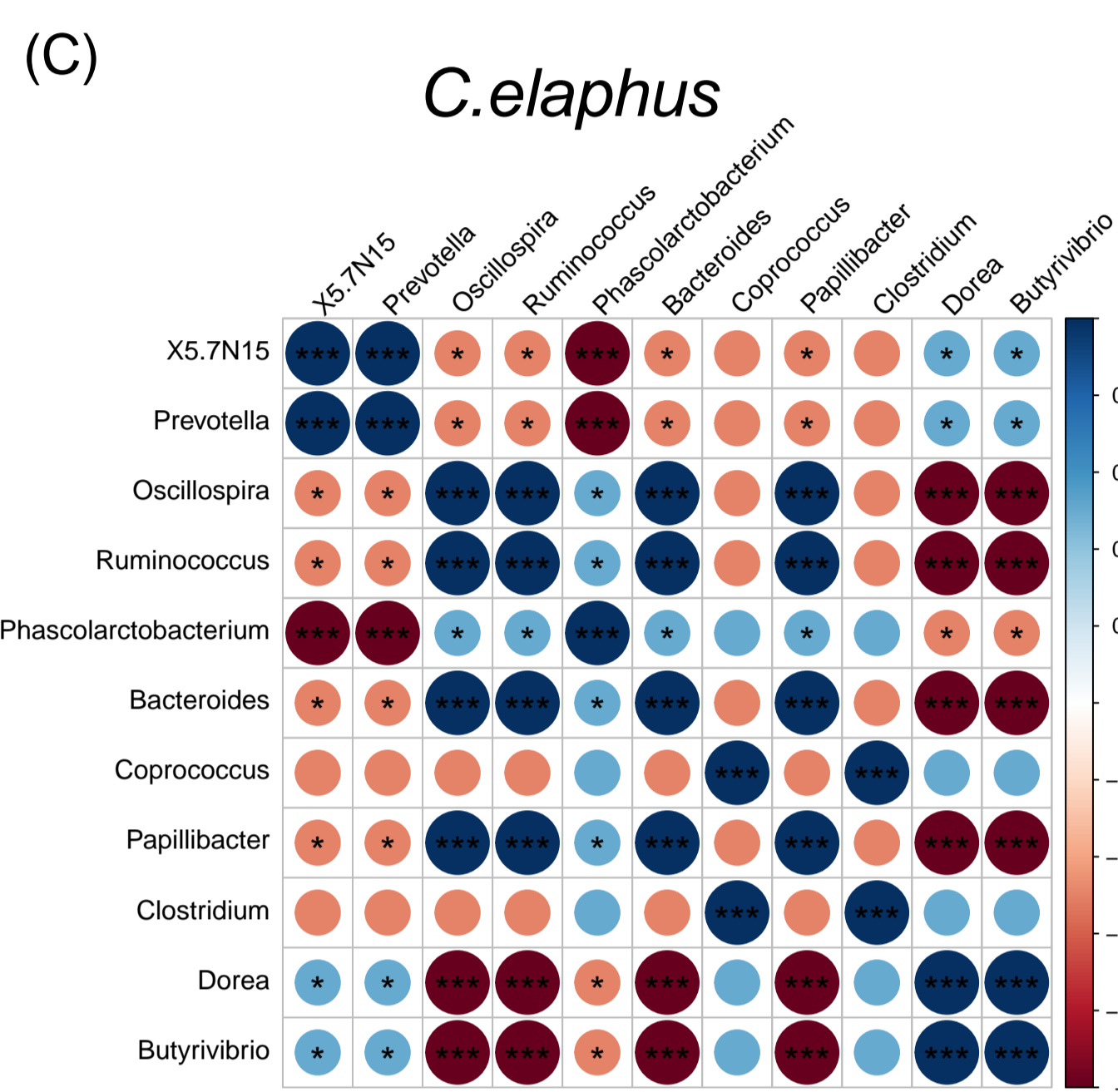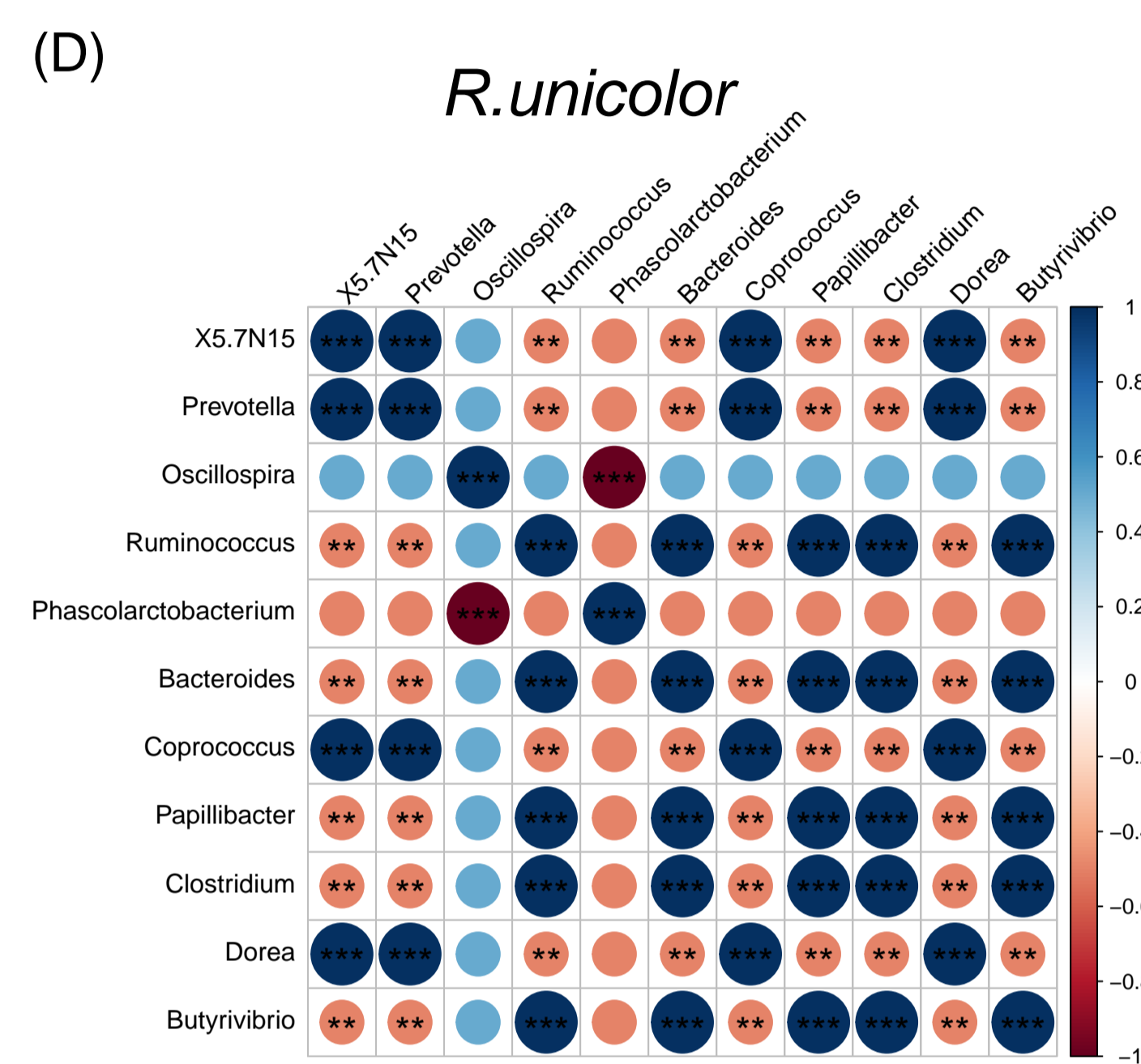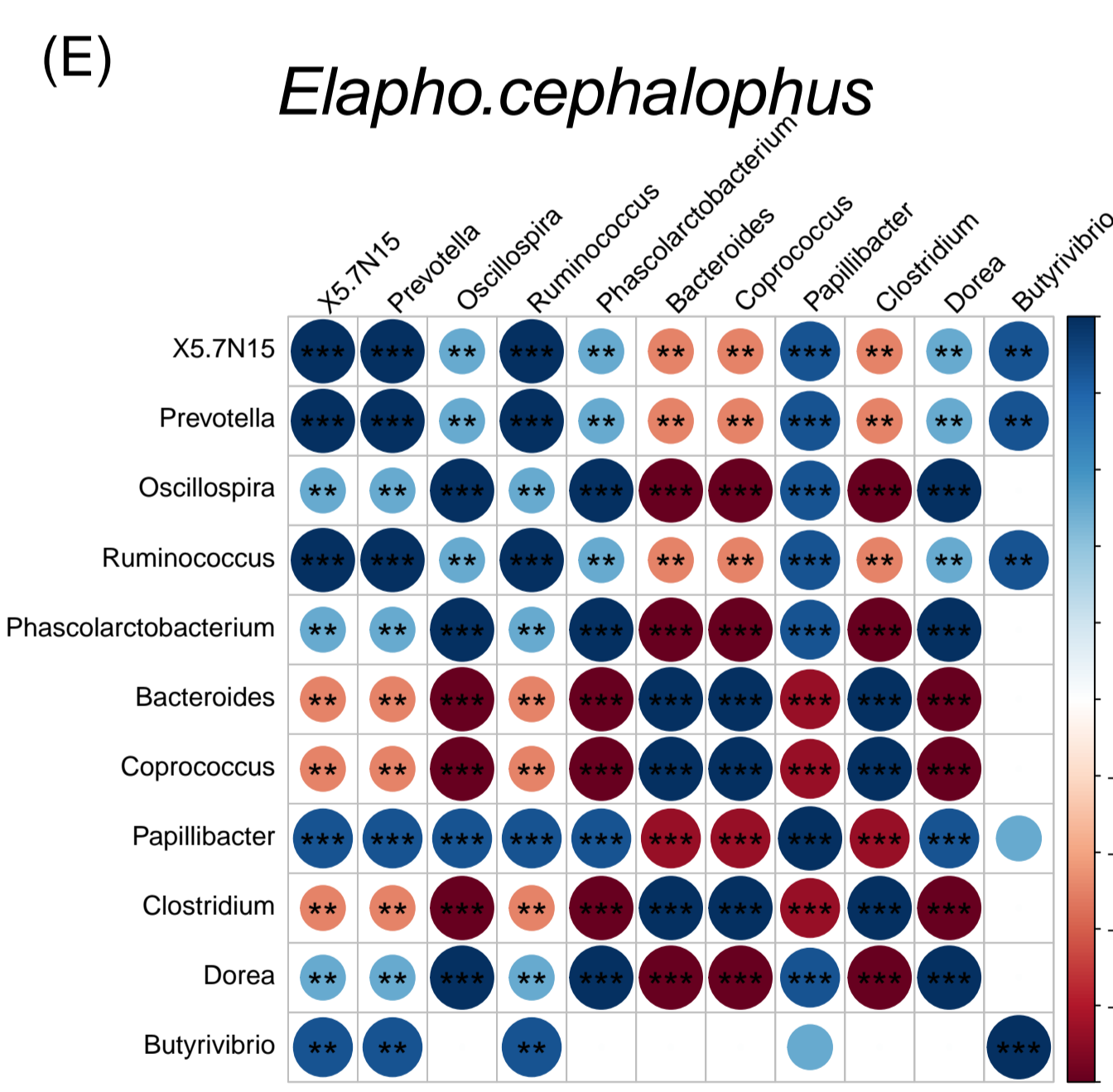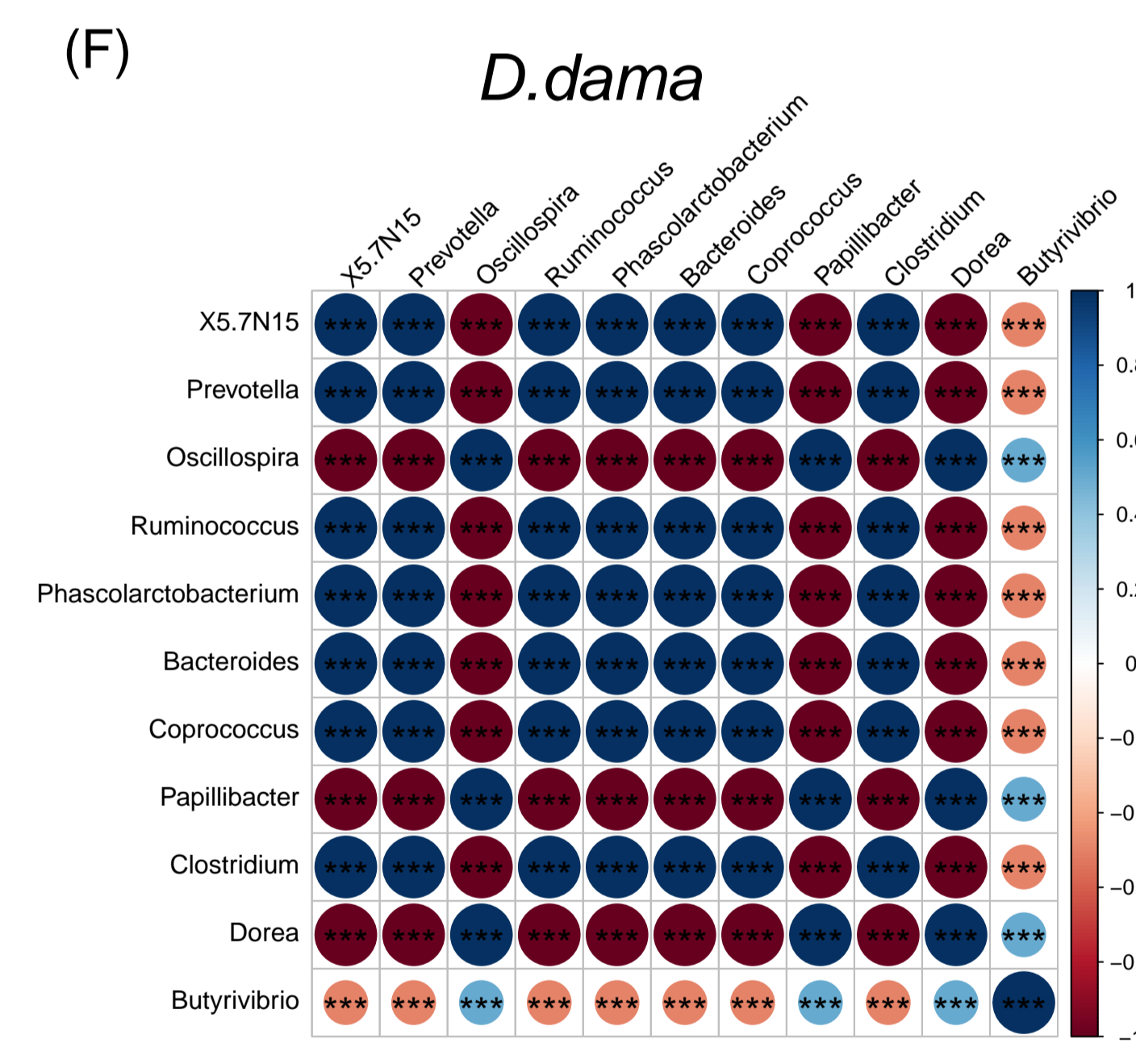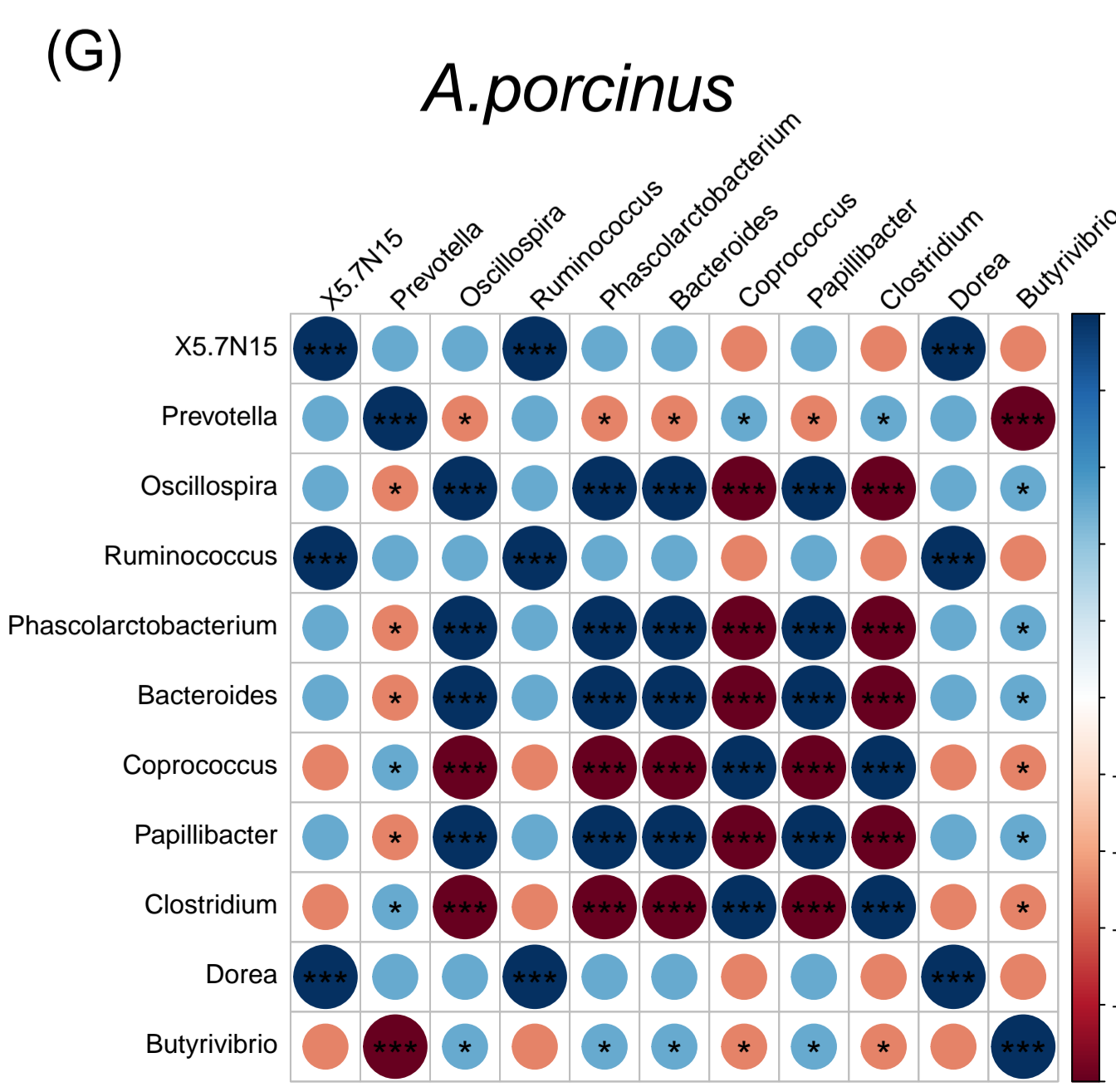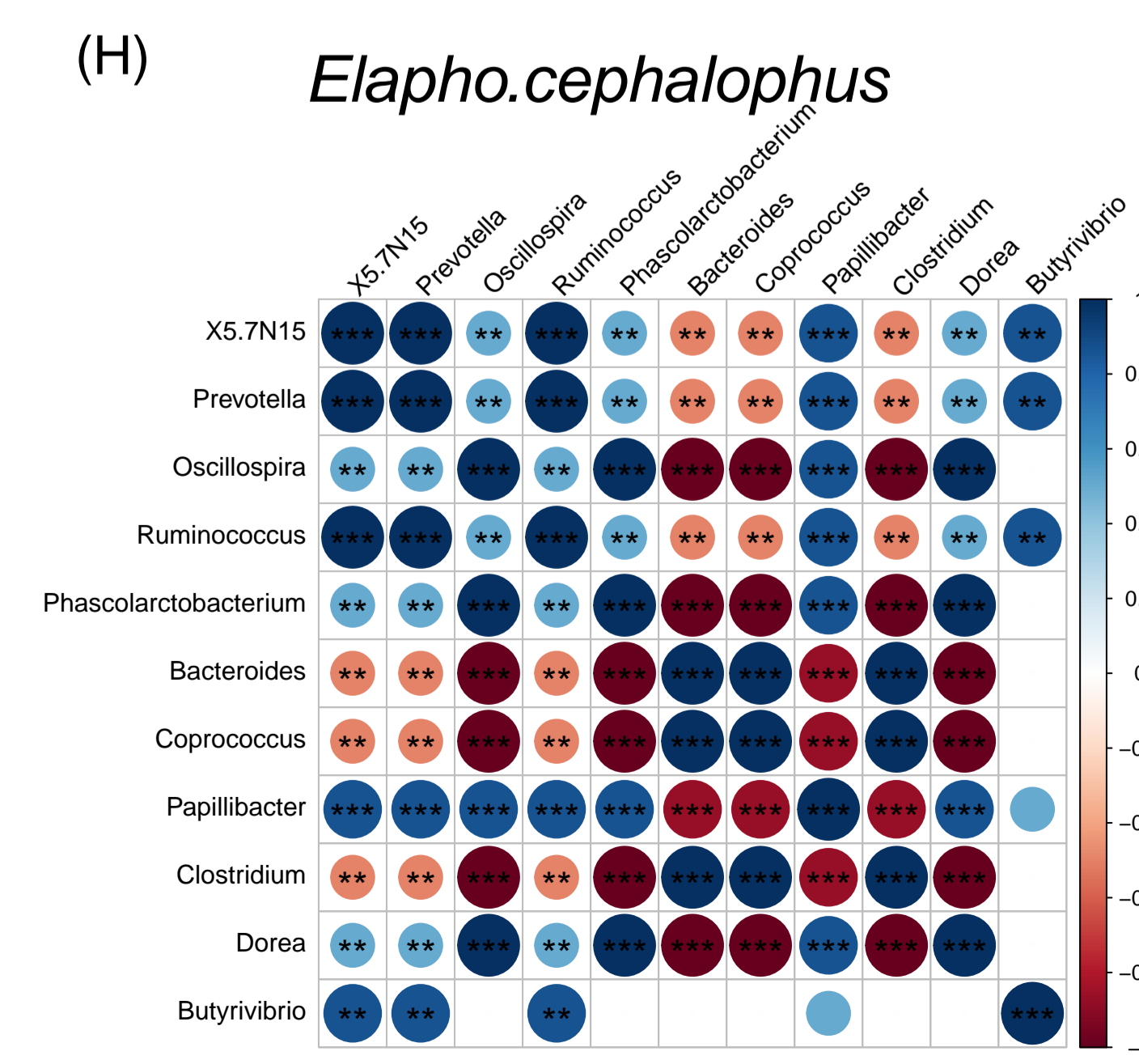

Supplement: FIGURE S1 — OTU-level rarefaction curves of Goods coverage across all samples. [file Data_Sheet_1.PDF]

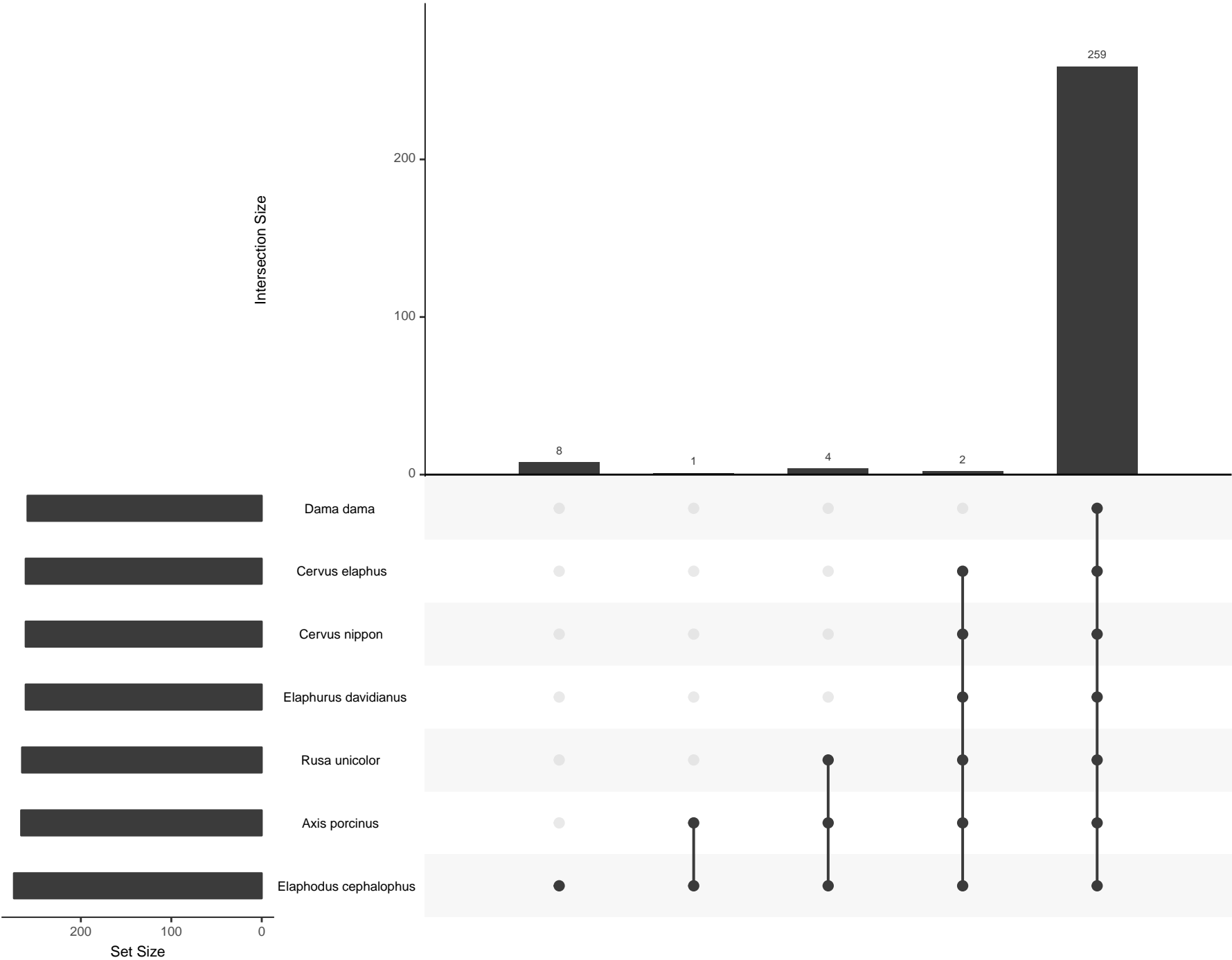

Supplement: FIGURE S2 — Co-occurrence patterns of core genera across the all host species (A) and within one host species (B–H), as determined by Spearman’s rank correlation analysis. [file Data_Sheet_2.PDF]

# BI tree

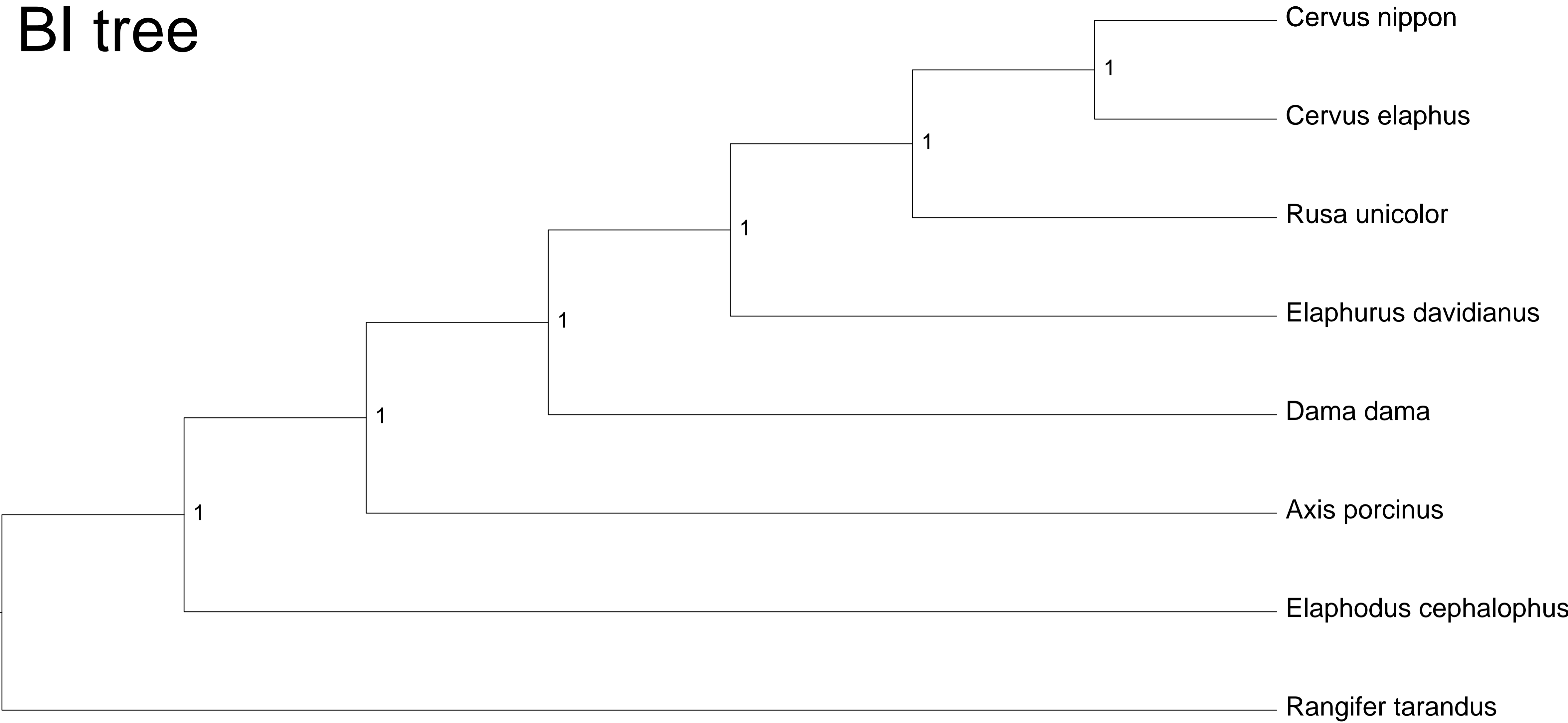

# ML tree

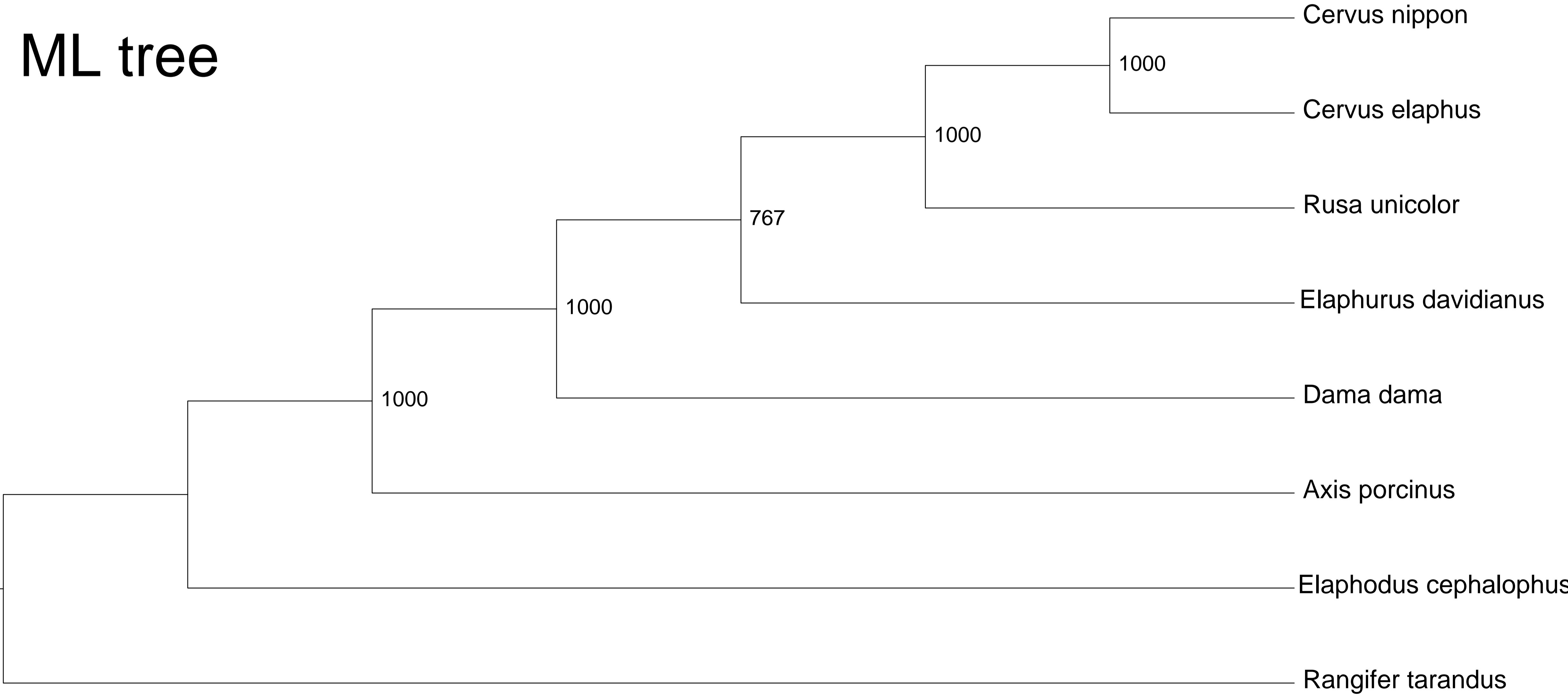

Supplement: FIGURE S3 — UpsetR plot of functional prediction. [file Data_Sheet_3.PDF]
